# Excess Entropy Scaling Law for Diffusivity in Liquid Metals

**DOI:** 10.1038/srep20689

**Published:** 2016-02-10

**Authors:** N. Jakse, A. Pasturel

**Affiliations:** 1 Sciences et Ingénierie des Matériaux et Procédés, UMR CNRS 5266, Grenoble Université Alpes, BP 75, 38402 Saint-Martin d’Hères Cedex, France

## Abstract

Understanding how dynamic properties depend on the structure and thermodynamics in liquids is a long-standing open problem in condensed matter physics. A very simple approach is based on the Dzugutov contribution developed on model fluids in which a universal (i.e. species-independent) connection relates the pair excess entropy of a liquid to its reduced diffusion coefficient. However its application to “real” liquids still remains uncertain due to the ability of a hard sphere (HS) reference fluid used in reducing parameters to describe complex interactions that occur in these liquids. Here we use ab initio molecular dynamics simulations to calculate both structural and dynamic properties at different temperatures for a wide series of liquid metals including Al, Au, Cu, Li, Ni, Ta, Ti, Zn as well as liquid Si and B. From this analysis, we demonstrate that the Dzugutov scheme can be applied successfully if a self-consistent method to determine the packing fraction of the hard sphere reference fluid is used as well as the Carnahan-Starling approach to express the excess entropy.

The study of the relationship between structural and dynamic properties of metallic liquids is of major importance since their coupling governs different processes like nucleation, crystal growth and glass-transition^1^,^2^,^3^,^4^. For a long time, theoretical developments of the liquid state dynamics have shown that dynamic properties can be determined from the knowledge of the static structure either directly in the framework of renormalized kinetic theories^5^,^6^,^7^,^8^,^9^ and early versions of the mode coupling theory^10^,^11^ (MCT) or through thermodynamic quantities like entropy in the Adam-Gibbs theory^12^. This line of thinking has been subsequently explored in several computational and experimental studies^13^,^14^,^15^,^16^,^17^,^18^,^19^. It has emerged from the latter the evidence of a strong connection between transport properties of fluids and their excess entropy, S_ex_, defined as the entropy of the liquid relative to that of the ideal gas at the same temperature and pressure. Such a correspondence is also of great practical value as it relates a dynamic quantity like the self-diffusion coefficient that can be difficult to measure accurately to an experimentally more accessible thermodynamic quantity. In addition, S_ex_, which can be expressed in terms of an expansion of *n*-body correlation functions, is often approximated by the first term, namely the two-body contribution S_2_. Indeed, it has been shown that S_2_ accounts for 80%–90% of the excess entropy for Lennard-Jones fluids^20^. In this case, S_2_ in unit of k_B_ can be written in terms of the pair-correlation function *g*(*r*) as


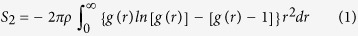


For model fluids, Rosenfeld was the first to show that transports properties like diffusivity and viscosity reduced by macroscopic thermodynamic properties (*e.g.* temperature, density) scale with the exponential of the excess entropy^13^,^14^. Dzugutov later recognized that diffusion is connected to atomic collisions with first neighbours, and more precisely to frequency of local structural relaxations. Then, he used these arguments to propose reduction parameters at the microscopic level within the hard sphere (HS) reference system, namely the particle diameter and effective Enskog interparticle collision frequency to justify a similar scaling law but using the two-body excess entropy only^15^. More recently, results based on the MCT provided a theoretical background to support the universal scaling law of Dzugutov for Lennard-Jones fluids^21^.

However, in spite of a large use of these entropy scaling laws, the precise origin of the apparent connection between thermodynamics and dynamics of fluids is still uncertain. For instance, for liquids metals modeled by more sophisticated potentials like EAM potentials, Hoyt *et al.*^22^ have pointed out the necessity to use a more exact excess entropy than the pair excess entropy in Dzugutov’s scaling law^15^. Moreover, these authors conclude that this scaling law does not hold for liquid silicon described by the Stillinger-Weber potential^23^ because the simple collision frequency term developed for hard spheres cannot capture correctly the local atomic structure of this element.

More generally, two main open questions are raised in the application of Dzugutov’s entropy scaling law to real liquids. What are the implications of the use of a HS reference system to describe the single-dynamics of a real liquid? Is the two-body approximation, S_2_, given by equation (1) sufficient to describe the excess entropy of a real liquid, since it is now well admitted^2^,^24^,^25^,^26^ that local structural ordering effects may be present in some of them?

Quite surprisingly, Dzugutov’s entropy scaling law has not been *hitherto* explored using *ab initio* molecular dynamics (AIMD) simulations which are in the mean-time known to provide a more powerful and realistic tool to investigate structural and dynamic properties of liquid metals^24^,^25^,^26^,^27^,^28^,^29^.

In this work, we propose to discuss the validity of the Dzugutov scheme when applied to real liquids by computing the diffusivities, radial distribution functions and excess entropies for various liquid metals, namely Al, Au, Cu, Li, Ni, Ta, Ti, Zn, using AIMD simulations. We also include in our study silicon and boron which are metallic in their liquid phases but display a more covalent local ordering^30^,^31^,^32^ in order to probe the universal character of these scaling laws for liquid metals.

## Results

### Diffusivity

**Figure 1:**
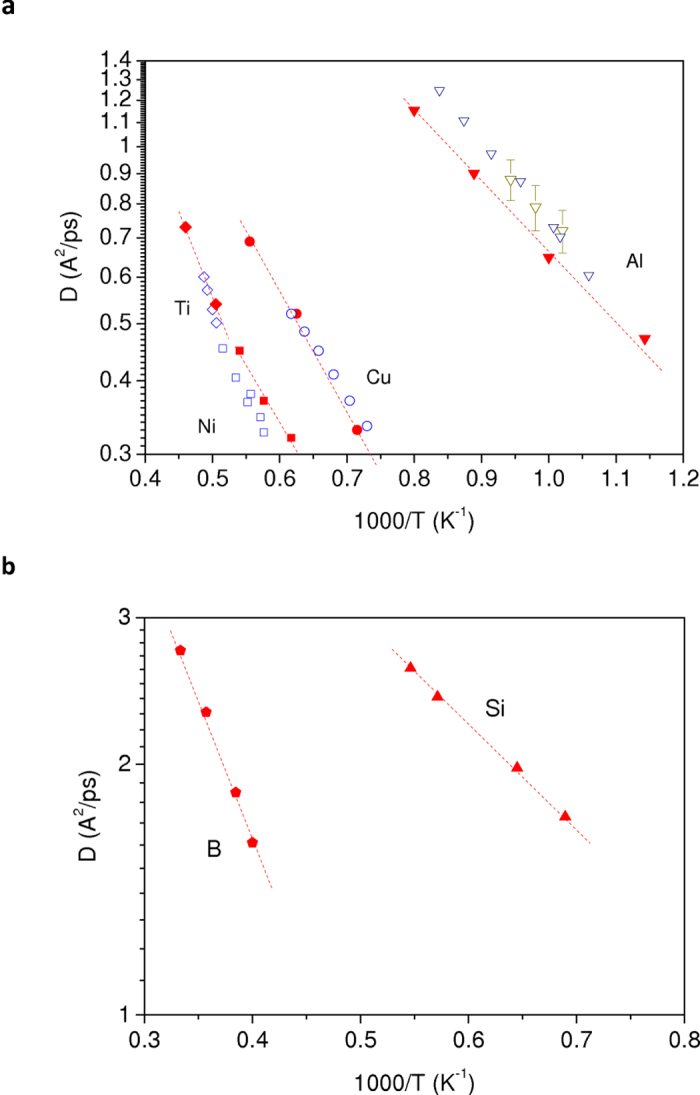
Temperature evolution of self-diffusion coefficients. (**a**) Calculated self-diffusion coefficients for aluminium (full triangle), copper (full circles), nickel (full squares) and titanium (full diamonds) are drawn as a function of inverse temperature. AIMD results are compared to the experimental data obtained by quasielastic neutron scattering for aluminium (open triangle), copper (open circles), nickel (open squares) and titanium (open diamonds) taken from refs. 10, 11, 12, 13. Calculated self-diffusion coefficients for Si (full triangle) and boron (full pentagons) are drawn as a function of inverse temperature. In both panels dashed lines are Arrhenius fits to AIMD results.

Firstly, we compute the temperature evolution of self-diffusion coefficients, *D*(*T*), of liquid metals through the time integration of the velocity auto-correlation function. This is equivalent to the determination of *D(T)* using the linear slope at long time of the mean-square displacement^33^, and requires shorter simulation times to have statistically meaningful results. We have checked for each element and for one temperature the consistency of the values of *D* obtained from both routes. The results do not depart more than 0.03 Å^2^/ps which guarantees the quality of the simulations performed here. In Fig. 1a, we compare our results obtained for Ni, Ti, Cu and Al to the most recent reliable experiments based on the use of quasielastic neutron scattering (QNS) *via* the determination of the incoherent intermediate scattering function^34^,^35^,^36^,^37^. We can see that AIMD calculations are in close agreement with experimental data. Moreover, we emphasize that the diffusion process in the liquid state of these different metals is characterized by an Arrhenius-type behaviour, which holds also for the more covalent liquid systems studied here, namely silicon and boron shown in Fig. 1b. These results suggest that the dynamics in these liquids is characterized by a homogeneous viscous flow which persists for all temperatures presented here. Let us mention that for metallic liquids in the deep undercooling regime, not studied in the present work, the particle motion becomes more complex with a non-Arrhenius behaviour, strong cooperative character and dynamical heterogeneity^38^.

### Pair correlation function

**Figure 2:**
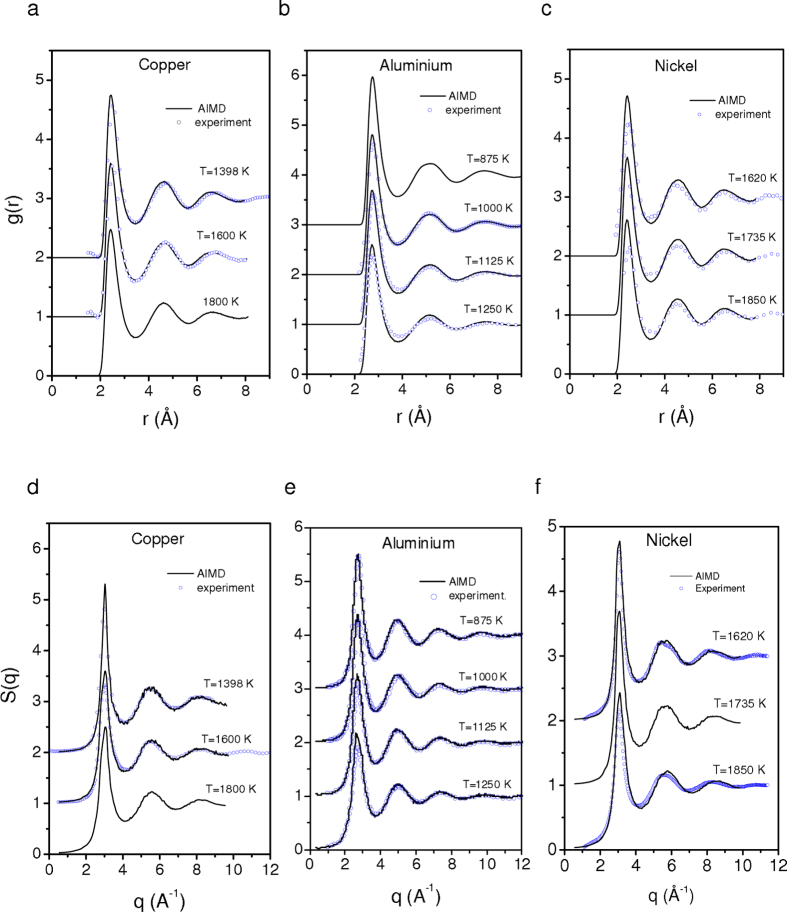
Temperature evolution of pair-correlation functions and structure factors from AIMD simulations. (**a**) Results for liquid copper at *T* = 1398, 1600 and 1800 K (full lines). Curves for *T* = 1398 and 1600 K are shifted upwards by an amount of 2 and 1, respectively. Open circles correspond to the x-ray diffraction data of ref. 14 (1398 K) and ref. 21a (1600 K). (**b**) Results for liquid aluminium at *T* = 875, 1000, 1125 and 1250 K (full lines). Curves for *T* = 875, 1000 and 1125 K are shifted upwards by an amount of 3, 2 and 1, respectively. Open circles correspond to the x-ray diffraction data of ref. 14 (1000 K) and ref. 20a (1125 and 1250 K). (**c**) Results for liquid nickel at *T* = 1620, 1735, and 1850 K (full lines). Curves for *T* = 1620 and 1735 K are shifted upwards by an amount of 2 and 1, respectively. Open circles correspond to x-ray diffraction values of Refs. 22a. (**d–f**) results of structure factors for copper, aluminium, and nickel, respectively, with the same captions as for pair-correlation functions.

An important structural quantity for the present work is the liquid structure for each metallic element that we have determined from AIMD simulations. We compare in Fig. 2 the calculated pair correlation function *g*(*r*) as well as static structure factor *S*(*q*) at different temperatures for Al, Cu, and Ni with corresponding measurements obtained *via* x-ray diffraction experiments^39^,^40^,^41^,^42^. Similar results for other metallic melts are presented in supplementary Figs 1, 2, 3, 4, 5, 6. A good agreement between simulated results and experimental data is achieved for all elements including Si and B liquids^30^,^31^,^32^. A small increase of the first peak of *g*(*r*) is detected upon cooling, while we do not find significant variation of the position of the first peak with a decrease of temperature, as reported recently by Lou *et al.*^43^. However, we note that this variation is very small and will not change the conclusions of our work. The coordination number in the first shell is obtained by integrating the first peak of *g*(*r*) up to its first minimum. We obtain an increasing trend with the decreasing temperature for all investigated elements. For Si and B liquids, we emphasize that our AIMD calculations lead to a fair agreement with experimental data and more particularly are able to describe the temperature evolution of the local structure marked by a reinforcement of the covalent character of bonds as the temperature decreases^30^,^31^,^32^.

### Diffusion and entropy scaling

With the results of the self-diffusion coefficients and pair-correlation functions obtained above from *ab initio* molecular dynamics simulations, we come now to the study of the diffusion and entropy scaling in the law proposed by Dzugutov^15^. It relates a dimensionless form of the diffusion coefficient, D^*^, to the pair excess entropy of the liquid phase, *S*_*2*_ as expressed in equation (1). The relationship can be written as:


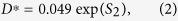


where D^*^ is obtained from the self-diffusion coefficient *D* using uncorrelated binary collisions described by the Enskog theory, namely:


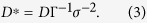


In equation (3), Γ is the Enskog collision frequency which can be calculated from the temperature *T* and number density ρ within the framework of the HS fluid:


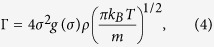


where *m*, ρ and σ are the atomic mass, atomic density and the hard-sphere diameter. The quantity *g*(σ) is the value of the HS pair-correlation function *g*(*r*) at the contact distance.

The first issue to address is the ability to use a HS reference fluid to describe the relationship between structural and dynamic properties of real liquids. More specifically, should one expect the self-diffusivity of a real liquid to be approximately equal to that of a HS fluid if compared at the same packing fraction 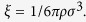 Note that this definition is a univocal link between the packing fraction and the HS diameter for a given number density of the real fluid.

Up to now, different approaches^22^,^44^ to test the scaling behavior for real liquids were based on the determination of pair-correlation functions from molecular dynamics that allow one not only to compute easily the *S*_*2*_ function but also to determine σ and *g(*σ) from the position and the height of the first peak of the pair correlation function. This method is the simplest way to determine the main parameters of the HS reference fluid.

**Figure 3:**
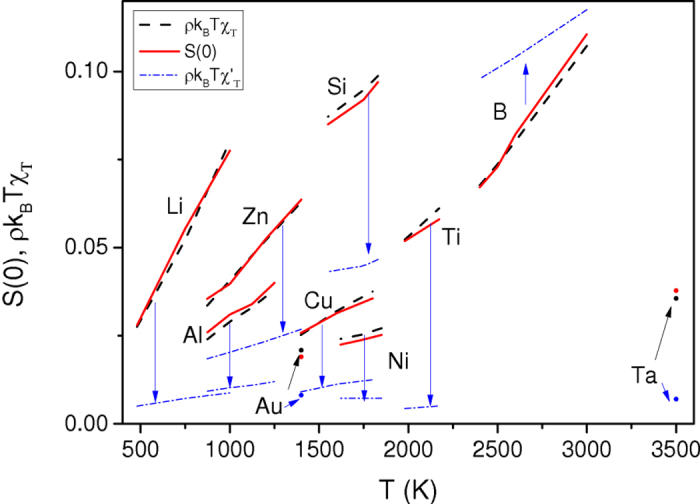
Thermodynamic consistency of the isothermal compressibility. Comparison of isothermal compressibility 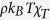 calculated from the Carnahan Starling expression (dashed lines) with that obtained from the extrapolation of *S*(*q*) at *q* = 0 (solid lines), with *S*(*q*) determined from *ab initio* calculations. The values of *S*(0) are obtained by a smooth extrapolation to *q* = 0 for all the elements by using a polynomial *S*(0) + *Aq*^2^ fitted on *S*(*q*) for *q* < 1.5 to determine parameters *A* and *S*(0) (ref. 48). Thin dashed dotted lines represent the isothermal compressibility 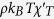 calculated from the Carnahan Starling expression using the packing fraction obtained from values of σ calculated from first peak position in AIMD pair-correlation functions (see text).

However, it is well known from perturbation theories^33^ that the effective HS diameter adjusted to represent the structure and thermodynamics of a real system is different from the simple choice for σ and *g*(σ) made above. In particular, this method leads to a lack of thermodynamic consistency between the HS reference fluid and the real one. To evidence this failure, we compare in Fig. 3 the isothermal compressibility calculated by the Carnahan Starling expression^45^, *i.e.* 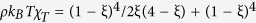, which is known to give a quasi-exact equation of states for HS fluids in a range of values of packing fraction characteristic of melts, with that obtained from the extrapolation of *S*(*q*) at *q* = 0, *S*(*q*) being the structure factor obtained from *ab initio* calculations. Fig. 3 indicates that the thermodynamic consistency, *i.e.*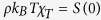, *via* calculations of the isothermal compressibility is not respected.

In this paper, we adopt a new approach to determine σ and *g*(σ) parameters. We keep the HS reference fluid but we enforce its thermodynamic consistency with the real liquid by imposing for each element that the values of σ and *g*(σ) satisfy the equality 
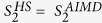
. 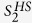 and 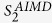 are calculated using equation (1) with the pair-correlation function obtained respectively from HS reference system and AIMD calculations. These values are then used to calculate D^*^ from equation (3) and (4), as well as the thermodynamically consistent value of the packing fraction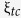 of the HS reference fluid.

To obtain the pair-correlation function of the HS model, 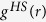, we use the integral equation (IE) method^46^, which is one of the most powerful semi-analytic method to determine *g*(*r*) of a fluid (see section Methods). The reliability of this approach is illustrated in Fig. 3, in which we can observe that the thermodynamic consistency *via* calculations of the isothermal compressibility is well respected with the values of σ and *g*(σ) optimized from the equality 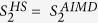.

**Figure 4:**
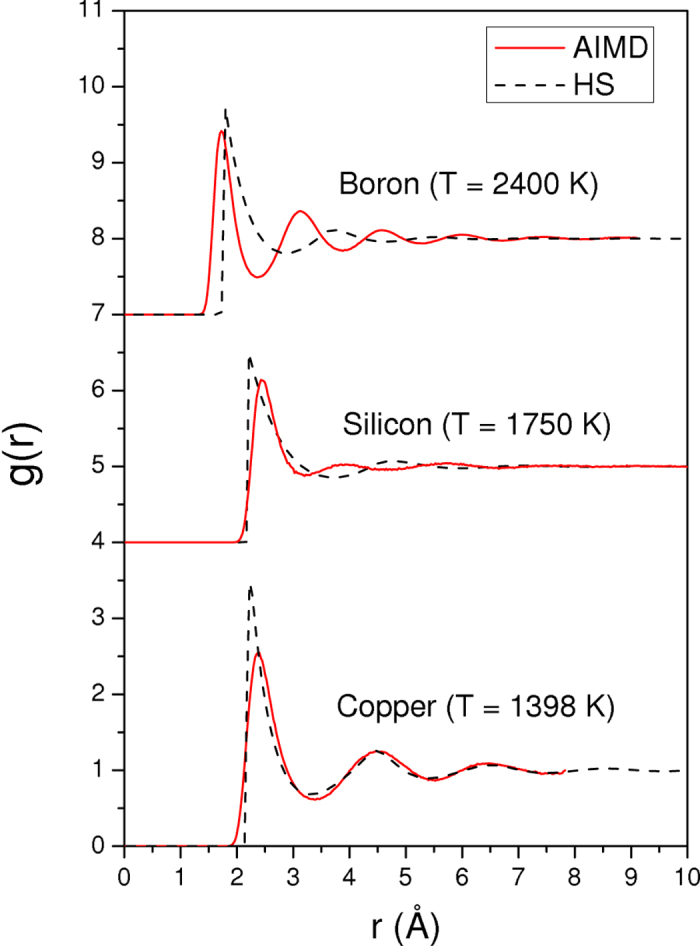
Pair-correlation functions of the hard-sphere reference fluid. Comparison of the hard sphere pair-correlation functions *g*(*r*), optimized using the self-consistent integral equation method, with results of AIMD simulations for copper (*T* = 1398 K), silicon (*T* = 1750 K) and boron (*T* = 2400 K). For the two latter elements the curves are shifted upwards by an amount of 4 and 7, respectively.

It is worthwhile to discuss the interest of our approach *via* the comparison of pair-correlation functions of the HS model obtained using the IE method with AIMD pair-correlation functions. In Fig. 4 we show this comparison for copper, which is representative of the other metallic elements studied here and not shown as well as for silicon and boron known to present a more covalent local ordering. For each element, our procedure leads to a good agreement for the first peak but one can notice that value of σ differs from that taken from the position of the first peak of *g*(*r*), leading to a packing fraction 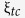
different from that obtained from the position of the first peak of the AIMD pair-correlation function. The same comment holds for *g*(σ) compared to the maximum of the AIMD pair-correlation function. Our results are similar to those found in the framework of perturbation theories^33^ within the optimized random phase approximation using a HS reference fluid^47^. For copper, we can observe that subsequent oscillations of the HS reference fluid are in phase with the AIMD curve while it is not the case for silicon and boron. This is not surprising given the complexity of the loosely packed structures found in liquid boron and silicon which cannot be fully accounted for by a HS fluid^48^.

Therefore choosing σ and *g*(σ) directly from AIMD pair-correlation functions is either not supported by this comparison and imposing the equality 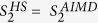 may circumvent the difficulty of the HS fluid to provide an accurate description of the local structural ordering of melts, especially for silicon and boron. Another important point is that a recent contribution has shown that this kind of fluids can still have a HS-like dynamics^49^.

## Discussion

We come now to the study of Dzugutov’s entropy scaling of the diffusion itself for all investigated elements.

**Figure 5:**
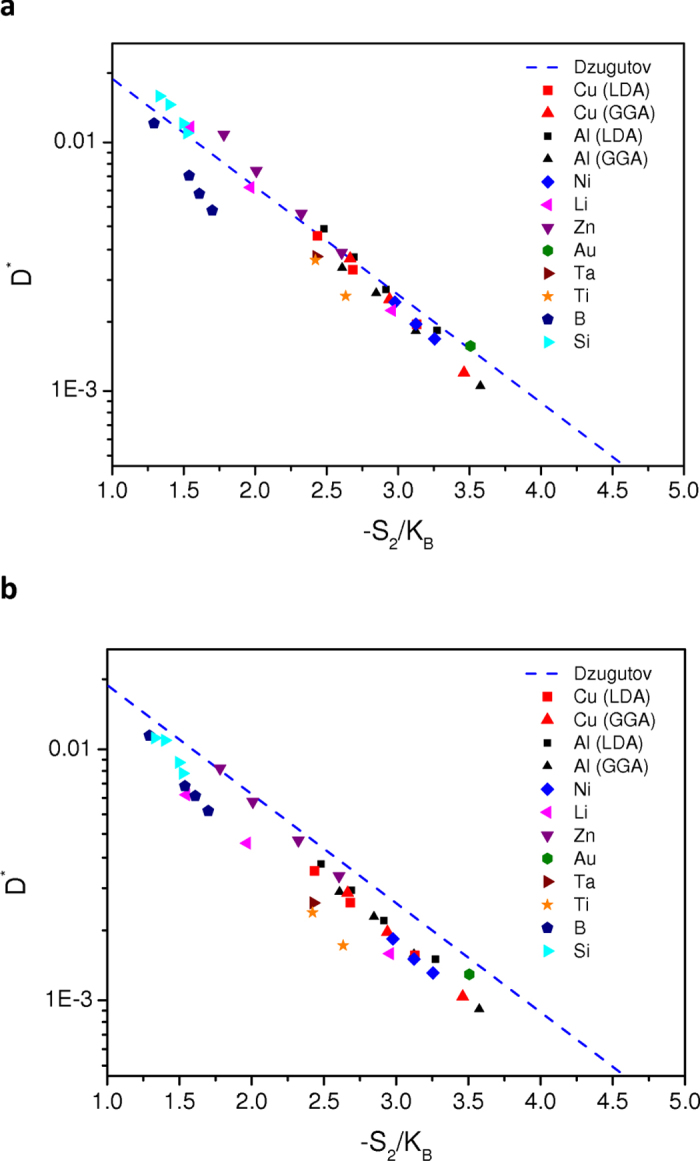
Dzugutov’s scaling law with two-body excess entropy. (**a**) Reduced diffusion D^*^ as a function of two-body entropy *S*_*2*_, with hard-sphere parameters σ and *g*(σ) obtained from the thermodynamically self-consistent integral equation scheme (see text) for all elements. (**b**) Same captions as for panel (**a**) but with σ and *g*(σ) determined from the position and height of first peak of the pair-correlation function calculated from *ab initio* molecular dynamics simulations.

In a first step, we plot in Fig. 5a the scaled diffusion coefficient D^*^ versus *S*_*2*_ with the values of σ and *g*(σ) optimized from the equality
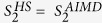
, and we compare our results with Dzugutov’s original scaling law given by equation (2). In Fig. 5b, we also report D^*^ determined this time with σ and g(σ) corresponding to the position and the height of the first peak in *g*(*r*) calculated from the AIMD simulations.

From comparison between Fig. 5a,b it is clear that the scaling law holds better when the IE method is used to determine σ and *g*(σ) parameters of the HS reference fluid. In this case, scatter in the data is smaller, and all the points are located closer to the original fit determined by Dzugutov (dashed line). In particular, liquid silicon is found to obey pretty well the scaling law. Our result is quite different from that obtained using the Stillinger-Weber potential^23^. In a very recent work^50^ however, substantial improvements have been obtained with the Stillinger-Weber potential when the total excess entropy is used rather than *S*_2_, for the stable liquid and moderate undercooling similar to the present study. It is worth mentioning that for deeper undercooling, below the maximum density temperature (*T* ~ 1350 K) corresponding also to a maximum of the excess entropy, it has been shown^50^ that an anomalous behavior with a different scaling law takes place. We can note that, as the temperature decreases, symbols representing the reduced diffusivity of boron are farther and farther from the dashed line. A close inspection indicates that this trend exists for all investigated elements with a slope higher than that of the original fit even if it is less pronounced than in the case of boron.

**Figure 6:**
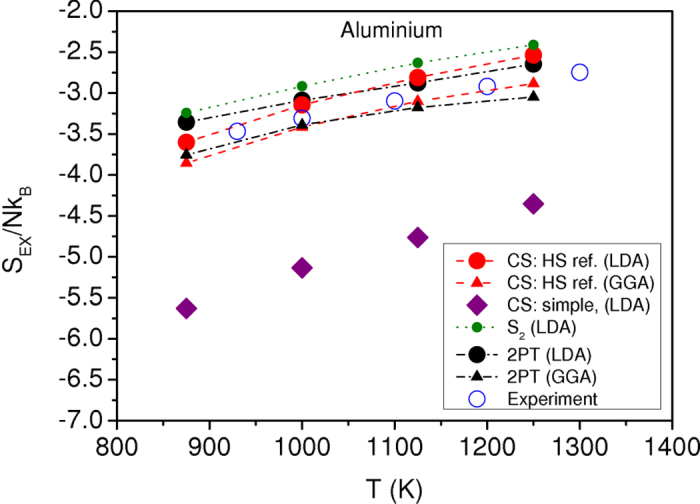
Temperature dependence of excess entropy of liquid aluminium. The Carnahan-Starling excess entropies given by equation (5), with hard-sphere parameters σ and *g*(σ) obtained from the thermodynamically self-consistent integral equation scheme, within the LDA (CS: HS ref. LDA) or the GGA (CS: HS ref. GGA) calculations are compared to the experiment of ref. 52. Within the LDA calculations, the excess entropies determined from the two-body approximation (*S*_2_ LDA) as well as the Carnahan-Starling expression given by equation (5) (CS: simple LDA), with hard-sphere parameter σ obtained from the position of the first peak of the AIMD pair-correlation function, are drawn. The excess entropy calculated from the 2PT model of ref. 54 is also shown for comparison.

In other words, the scatter exhibited in Fig. 5a may origin in the fact that the *S*_*2*_ is not completely reliable for real systems studied using AIMD calculations and in particular is not enough accurate to describe the temperature dependence of D^*^. As already discussed, it is known that for model fluids, the pair entropy accounts for 80%–90% of the excess entropy^20^. But certainly, the key point is that *S*_*2*_ has been recently shown^51^ to display slightly different temperature dependence from that of *S*_*ex*_ (see also Fig. 6).

Therefore, we can address the second issue and test the scaling law involving a more reliable determination of *S*_*ex*_ but still keeping the framework of the HS reference fluid. It is based on the Carnahan-Starling equation^45^, which is known to give values of entropies of liquid metals at the melting point in close agreement with experimental ones^48^. In this formalism, the excess entropy can be written as:


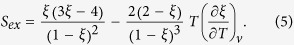


The merit of this approach is an analytical description of the excess entropy which has also an explicit temperature dependent part. Note that this temperature dependence has been shown to be essential to describe thermodynamic properties of liquid metals like their entropies or their heat capacities at their melting temperature^48^. We can add that the evolution of the packing fraction with temperature is given mainly by the evolution of the experimental density with temperature since the temperature dependence of *g*(*r*) and then σ is small in our simulations.

Taking advantage of the simple analytical expression given by Eq. (5), we calculate the Carnahan-Starling excess entropy for each element using the thermodynamically consistent values of the packing fraction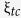. In Fig. 6, the obtained values are displayed for liquid Al as a function of temperature for the LDA and GGA *ab initio* simulations, and compared to the experimental data of Hultgren *et al.*^52^. A good agreement is seen, with only a slight overestimation for the LDA and a slight underestimation for the GGA. Such a result indicates the reliability of the approach propounded in the present study. As mentioned above, we can be seen in Fig. 6 that *S*_2_ values overestimate the Carnahan-Starling ones by about 10%, and display a weaker slope. We have also drawn the Carnahan-Starling excess entropy, but using the packing fraction determined with σ corresponding to the height of the first peak of the AIMD pair-correlation function. This simple choice of σ leads to a significant underestimation of the excess entropy, and would lead to a significant departure from Dzugutov’s scaling law. This is a direct consequence of the fact that the thermodynamic consistency is not respected in this case, as for isothermal compressibility shown in Fig. 3.

A recent work^53^ has shown that an accurate estimate of the excess entropy for liquid metals can be obtained using a two phase thermodynamic (2PT) model^54^ developed on dynamic grounds, in which the vibrational density of states (the Fourier transform of the velocity auto-correlation function) is decomposed on solid and gas phase parts. In Fig. 6, we draw the curves of the excess entropy for liquid Al estimated from the 2PT model for the LDA and GGA simulations. The curves are close to the corresponding Carnahan-Starling excess entropy which shows again the reliability of our method. We note however that the 2PT curves show weaker temperature dependence than the Carnahan-Starling excess entropy.

**Figure 7:**
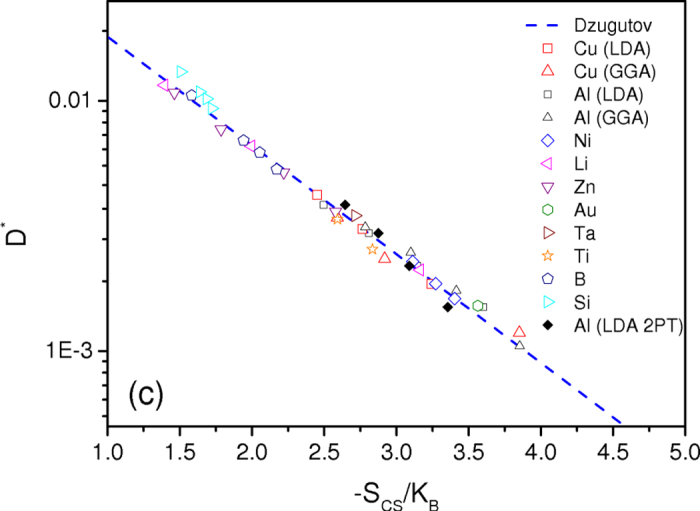
Dzugutov’s scaling law with Carnahan and Starling excess entropy. Reduced diffusion as a function of excess entropy *S*_CS_ for all elements. Hard-sphere parameters σ and *g*(σ) are obtained from the thermodynamically self-consistent integral equation scheme (see text) for all elements, and *S*_CS_ is determined from the Carnahan and Starling expression, given by equation (5). Note that for Al and Cu liquids, we performed two series of calculations to check that GGA and LDA exchange-correlation functionals used in AIMD calculations do not modify the main conclusions of our discussion. The reduced diffusion as a function of excess entropy calculated from the 2PT model is also shown for liquid Al within the LDA.

Finally, we plot in Fig. 7 the relationship between the excess entropy and the dimensionless form of the diffusion coefficient, D^*^, for all the investigated elements. It is found that the scaling law proposed by Dzugutov is legitimate since, with the use of the excess entropy derived here from the Carnahan-Starling approach, there appears to be less scatter in the data and the linearity with temperature for a given metallic element is obeyed very well. We observe that Dzugutov’s law is equally well obeyed with the 2PT model, with a slightly different slope that can be attributed to the temperature dependence of the excess entropy thus obtained. We also conclude that results for liquid silicon and boron are still acceptable emphasizing the ability of this new formulation to treat loosely packed and more complex structures which may occur in some real liquids^55^. How this formulation can be applied to liquid alloys is an important open question to be addressed in future work.

## Methods

### *Ab initio* molecular dynamics

The AIMD simulations were performed within the framework of plane-wave-based density-functional theory (DFT) using the Vienna *ab initio* simulation package^56^. A plane-wave basis set with a specific energy cutoff is used for each element as given in the Supplementary Information. The electron-ion interaction was described by the projected augmented-wave method^57^,^58^, and either the local-density approximation^59^ or the generalized gradient approximation (GGA) in the Perdew-Burke-Ernzerhof (PBE) form^60^,^61^ for the exchange-correlation energy depending on the element considered. Note that for Al and Cu, both GGA and LDA were investigated to test their influence on the results.

All the dynamical simulations were carried out in the NVT ensemble by means of a Nosé thermostat to control temperature. Newton’s equations of motion were integrated using Verlet’s algorithm in the velocity form with a time step of 1 fs. A cubic unit cell with periodic boundary conditions containing 256 atoms was used in the simulation. Only the Γ-point sampling was considered to sample the supercell Brillouin zone.

The liquid samples were first prepared at a temperature well above the highest one studied to reach thermal equilibrium. This was followed by a cooling to the successive lower desired temperatures for the given element with a rate of 10^13^ K/s. At each temperature, the volume *V* of cell was chosen to reproduce the experimental densities (see the Supplementary Information). After an equilibration for 3 ps at the studied temperature, the run was continued for 80 ps. A number of 1000 configurations were collected during this time to produce averaged quantities, namely the pair-correlation function and static structure factor by standard techniques^33^,^62^,^63^.

For the dynamics, we focus on the self-diffusion coefficients are characterized by the individual atom displacement through the mean-square displacement^62^,^63^ (MSD)


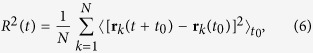


where 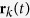 is the position of atom *k* at time *t*, and angular brackets represent the average over time origins *t*_*0*_. The self-diffusion coefficient *D* can be determined from the long time slope of 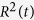, namely:


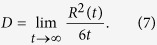


Alternatively *D* can be calculated from the Green-Kubo time integral of 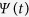



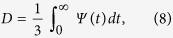


where


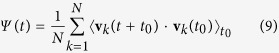


is the velocity auto-correlation function obtained from the velocity 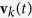 of atom *k* at time *t*. In the present work, we use the velocity autocorrelation to determine *D*.

### Integral equation method

The computation of the pair-correlation function from the hard-sphere potential is done by the integral equation method, using as an input only the packing fraction 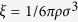, where 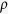 is the experimental density of each element. This method is based on the exact Ornstein-Zernike convolution equation:


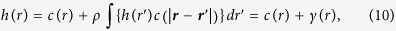


where *h*(*r*), *c*(*r*) and 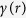 are respectively the total, direct and indirect correlation functions between two atoms, separated by a distance *r* in a liquid composed of *N* atoms with number density 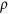
33. In order to determine the pair-correlation function *g*(*r*) = *h*(*r*) + 1, this equation has to be solved together with a closure relation whose formal expression is


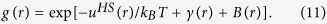


In this equation, 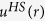 is the hard-sphere potential and 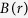 is the so-called bridge function composed of an infinite series of elementary bridge diagrams^64^. 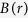 is obtained using the efficient approximate formulation for the hard-sphere model proposed by Rodgers and Young^65^:


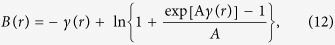


which guarantees a very accurate description of thermodynamic properties of the reference fluid through the optimization of the parameter A^66^. This set of equations are solved numerically^67^,^68^ by using the method originally proposed by Gillan^69^ and improved later by Labik *et al.*^70^. It combines the Newton-Raphson (NR) algorithm and the successive substitution method by introducing a sine basis functions. This method is known as very fast and stable.

From a technical point of view, the correlation functions are discretized on a grid of *N* = 2048 points with a regular mesh of *r* = 0.025σ, which represent an extension of the correlation functions in the real space of 51.2σ. Such a choice is sufficient to get accurate results whatever the thermodynamic state point^71^. When a solution cannot be found, due for instance to the lack of convergence or singularity of the linearized set of equations, controls on iteration number and singularity were added to leave the NR algorithm without stopping the adaptive scheme to reach the desired state point. Moreover, we apply the tangent linear differentiation technique that is an essential ingredient to improve the numerical procedure of the self-consistent integral equation method. We refer the reader to refs. 67 and 68 for a more detailed description of the method.

## Additional Information

**How to cite this article**: Jakse, N. and Pasturel, A. Excess Entropy Scaling Law for Diffusivity in Liquid Metals. *Sci. Rep.* **6**, 20689; doi: 10.1038/srep20689 (2016).

## Supplementary Material

Supplementary Information
