# Supplementary material for: Excess Entropy Scaling Law for Diffusivity in Liquid Metals
Source: Sci Rep. 2016 Feb 10;6:20689. doi: 10.1038/srep20689 (PMC4748237; doi:10.1038/srep20689)
Supplement: Supplementary Information [file srep20689-s1.pdf]

Excess Entropy Scaling Law for Diffusivity in Liquid Metals

N. Jakse and A. Pasturel

Sciences et Ingénierie des Matériaux et Procédés, UMR CNRS 5266, Grenoble Université Alpes, BP 75, 38402 Saint-Martin d'Hères Cedex, France

Figures

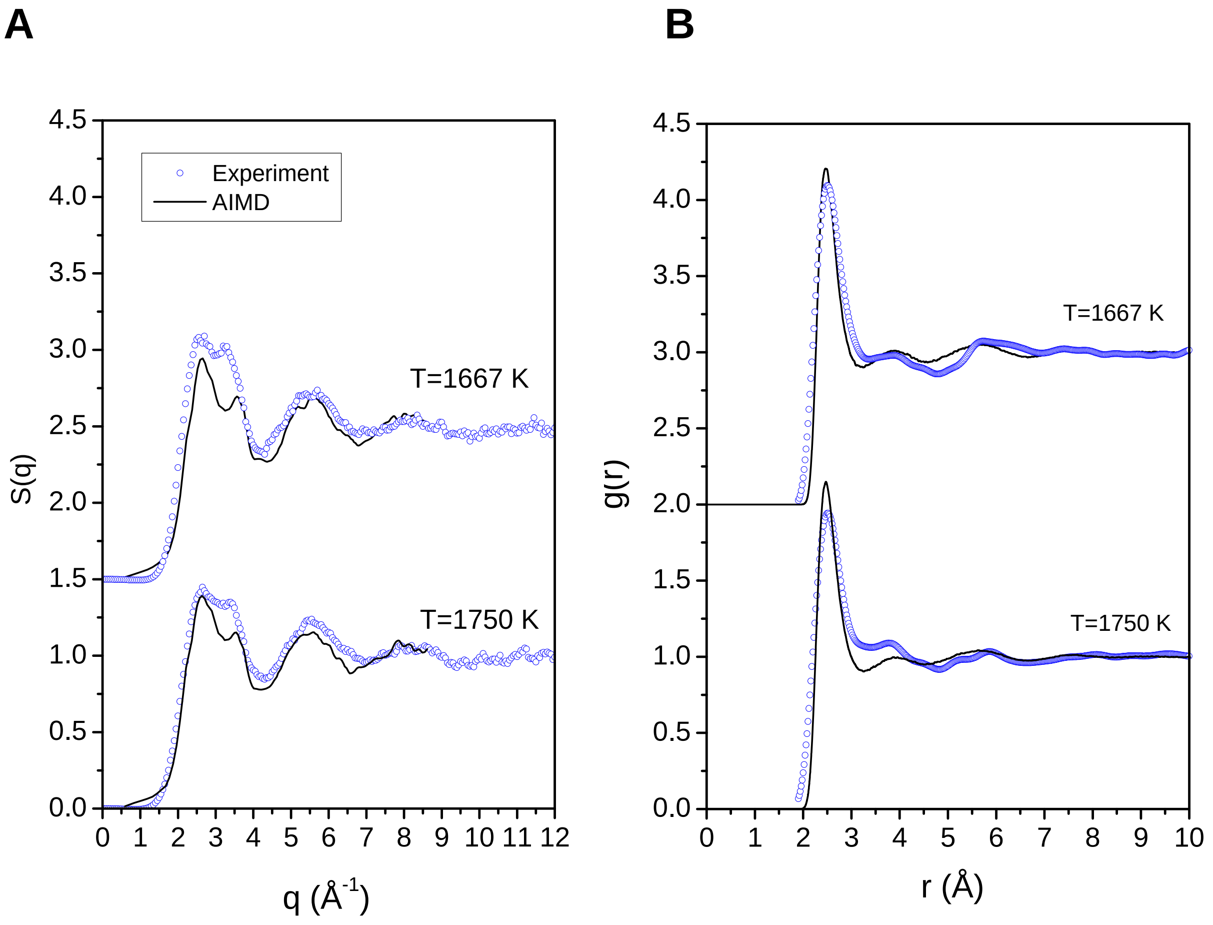

Fig S1. Structure factors (A) and pair-correlation functions (B) of liquid Si at T = 1750 K and 1667 K. AIMD results of this work (lines) are compared to the x-ray measurements (open circles) of Jakse *et al.* (Ref. S1). Curves at T = 1667 K are shifted for clarity by an amount of 1.5 for $S(q)$ and 2 for $g(r)$.

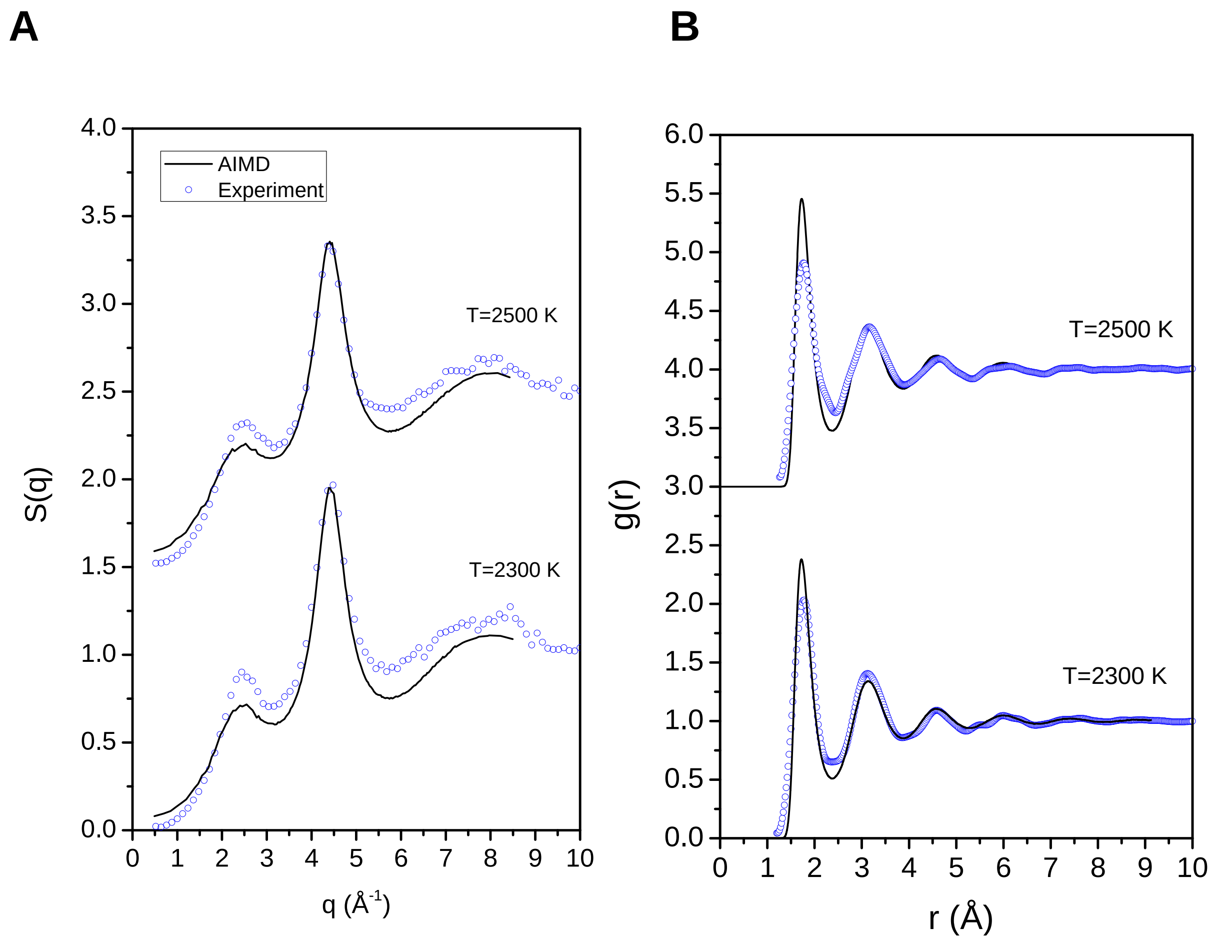

Fig S2. Structure factors (A) and pair-correlation functions (B) of liquid B at T = 2300 K and 2500 K. AIMD results (solid lines) are compared to the x-ray measurements (open circles) of Price *et al.* (Ref. S2). Curves at T = 2500 K are shifted for clarity by an amount of 1.5 for $S(q)$ and 3 for $g(r)$.

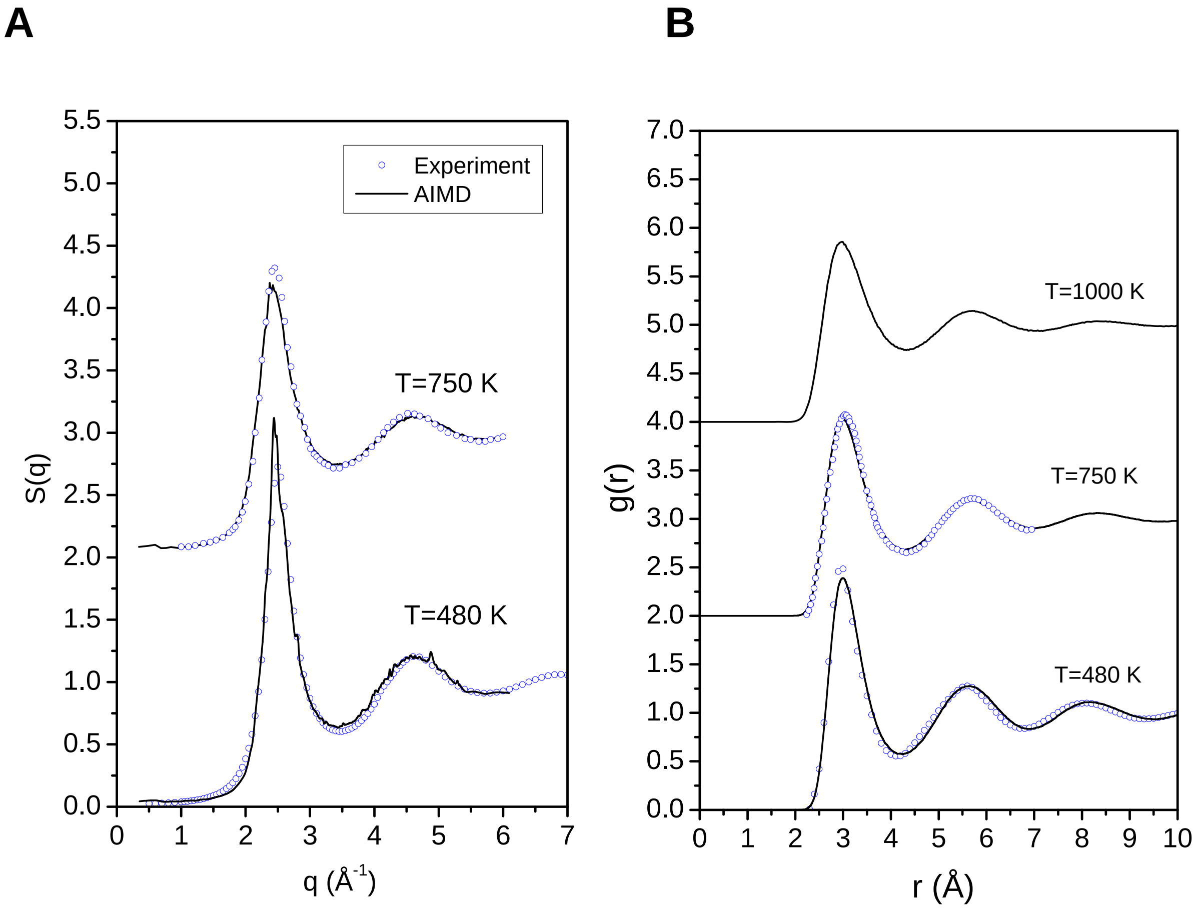

Fig. S3. Structure factors (A) and pair-correlation functions (B) of liquid Li at T = 480 K, 750 K and 1000 K for g(r). AIMD results (solid lines) are compared to the x-ray measurements (open circles) of Waseda (Ref. S3) at T = 480 K and neutron diffraction measurements of Olbrich *et al.* (Ref. S4). Curves at T = 750 K are shifted for clarity by an amount of 2 for $S(q)$ and 2 for $g(r)$. The curve of $g(r)$ at 1000 K is shifted by an amount of 4.

A **B**

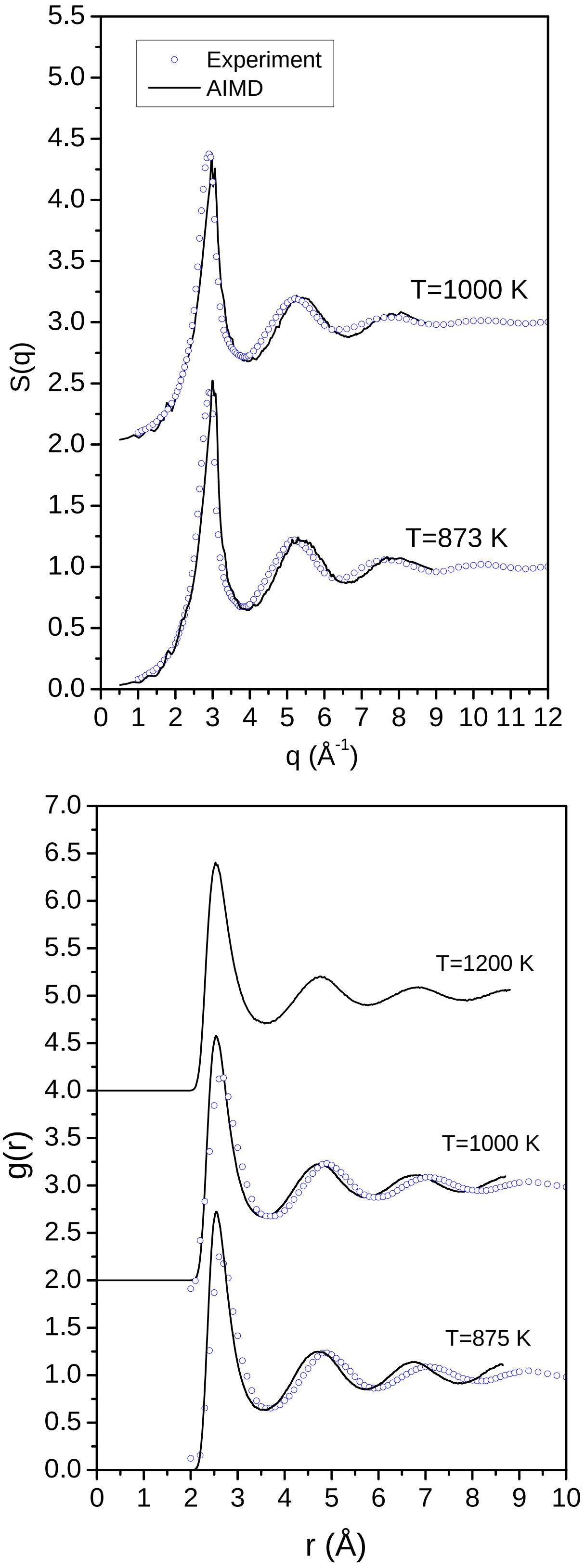

Experiment
AIMD
T=1000 K
T=873 K
S(q)
q (Å⁻¹)
T=1200 K
T=1000 K
T=875 K
g(r)
r (Å)

Fig. S4. Structure factors (A) and pair-correlation functions (B) of liquid Zn at T = 873 K, 1000 K and 1200 K for g(r). AIMD results (solid lines) are compared to the x-ray measurements (open circles) of Waseda (Ref. S3). Curves at T = 1000 K are shifted for clarity by an amount of 2 for $S(q)$ and for $g(r)$. The curve of $g(r)$ at 1200 K is shifted by an amount of 4.

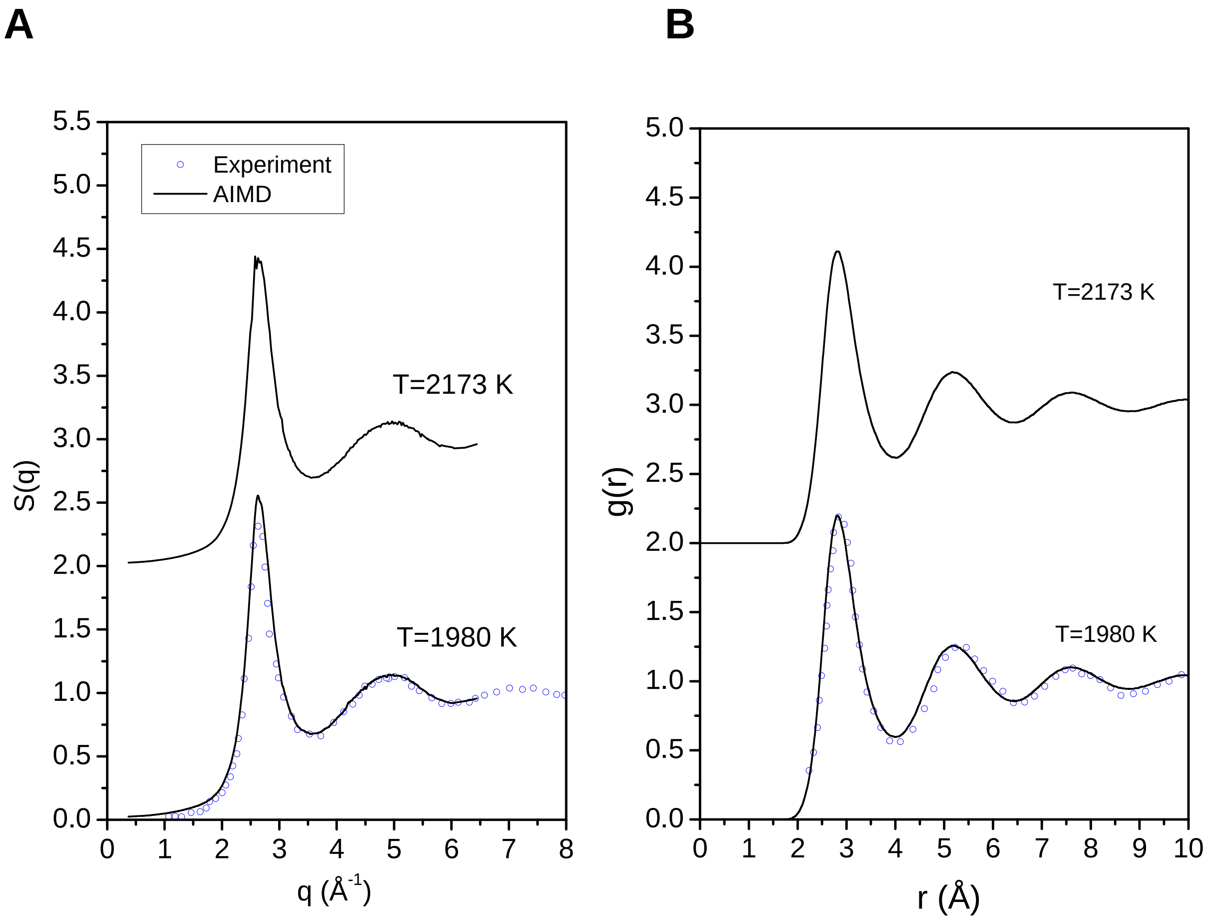

Fig. S5. Structure factors (A) and pair-correlation functions (B) of liquid Ti at T = 1980 K and 2173 K. AIMD results of this work (solid lines) are compared to the x-ray measurements (open circles) of Holland-Moritz *et al.* (Ref. S5) at T = 1980 K. Curves at T = 2173 K are shifted for clarity by an amount of 2 for $S(q)$ and for $g(r)$.

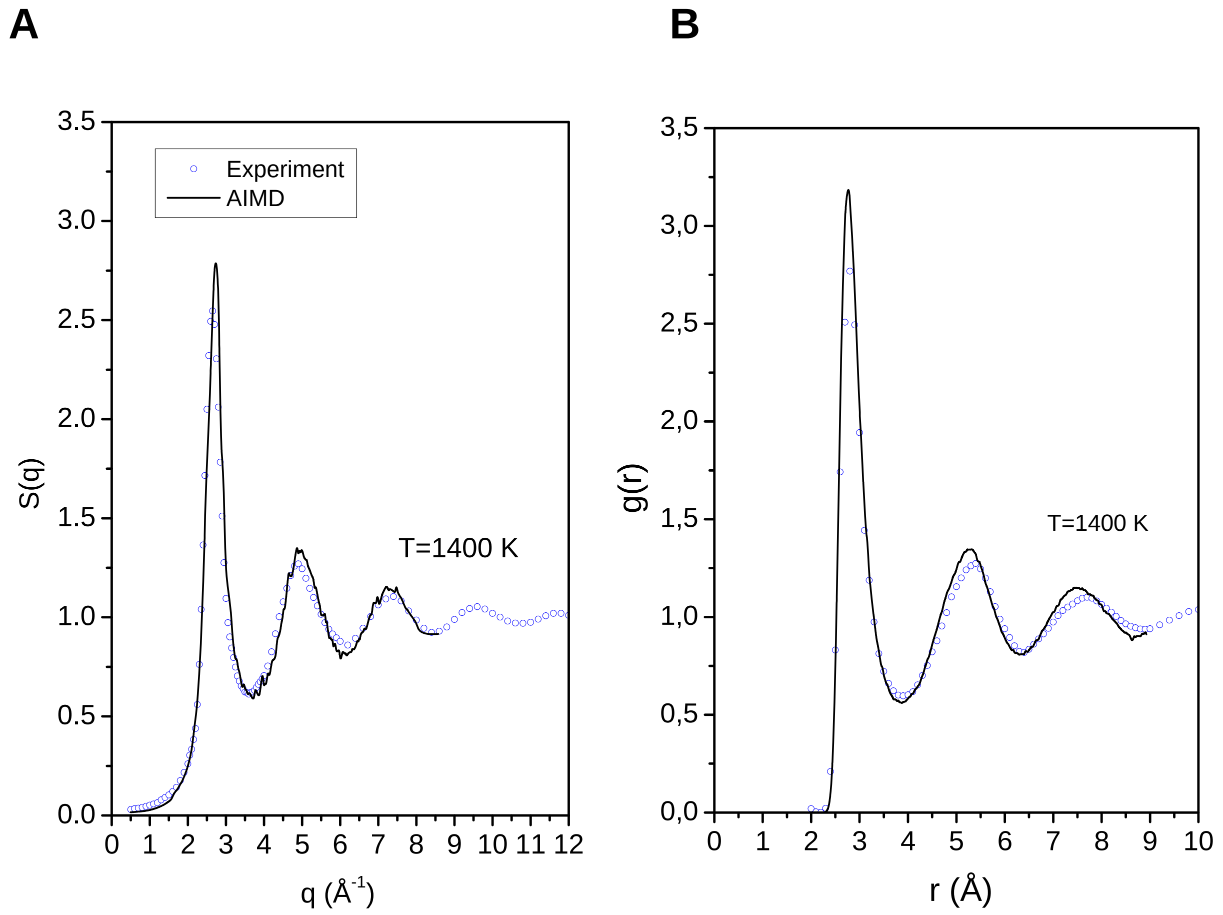

Fig. S6. Structure factor (A) and pair-correlation function (B) of liquid Au at T = 1400 K. AIMD results of this work (solid lines) are compared to the x-ray measurements (open circles) of Waseda (Ref. S4).

Table

	T (K)	ρ (A^{-3})	E_{cut} (eV)
Al	850<br>1000<br>1125<br>1250	0.0535<br>0.0526<br>0.0520<br>0.0512	241
Li	480<br>750<br>1000	0.0438<br>0.0415<br>0.0403	140
Zn	873<br>1000<br>1200<br>1400	0.0590<br>0.0578<br>0.0560<br>0.0542	277
Cu	1398<br>1600<br>1800	0.0758<br>0.0730<br>0.0716	273
Au	1400	0.0529	230
Ni	1620<br>1735<br>1850	0.0811<br>0.0805<br>0.0800	270
Ti	1980<br>2173	0.0602<br>0.0543	179
Ta	3500	0.0499	224
Si	1550<br>1667<br>1750<br>1832	0.0551<br>0.0541<br>0.0535<br>0.0530	245
B	2300<br>2400<br>2500<br>3000	0.1246<br>0.1231<br>0.1218<br>0.1204	319

Table S1. Parameters of the AIMD simulations: Temperatures T and experimental densities ρ as well as the energy cutoff E_{cut} used in the AIMD simulations are given for all the elements. Densities are taken from the following references: Al (Ref S6), Li (Ref. S7), Zn (Ref. S7), Cu

(Ref. S8), Au (Ref. S9), Ni (Ref. S10), Ti (Ref.S11), Ta (Ref. S12), Si (Ref. 13), and B (Ref. S14).

Supporting References

1. Jakse N, Hennet L, Price DL, Krishnan S, Key T, Artacho E, Glorieux B, Pasturel A, Saboungi ML (2003) Structural changes on supercooling liquid silicon. *Appl. Phys. Lett.* **83**, 4734–4736.

2. Price DL, Alatas A, Hennet L, Jakse N, Krishnan S, Pasturel A. Pozdnyakova I, Saboungi ML, Said A, Scheunemann R, Schirmacher W, Sinn H (2009) Liquid boron: X-ray measurements and ab initio molecular dynamics simulations. *Phys. Rev. B* **79**, 134201.

3. Waseda Y (1980) *The Structure of Non-Crystalline Materials* (Mc Graw-Hill, New York). http://res.tagen.tohoku.ac.jp/~waseda/scm/LIQ/, 01/02/2013, Structural Characterization of Materials Liquid Database, Institute of Advanced Materials Processing, Tohoku University, Japan.

4. Salmon PS, Petri I, de Jong, PHK, Verkerk P, Fischer HE, Howells WS Structure of liquid lithium. (2004) *J. Phys.: Condens. Matter* **16**, 195–222.

5. Holland-Moritz D, Heinen O, Bellissent R, Schenk T (2007) Short-range order of stable and undercooled liquid titanium. *Mat. Sci. Eng. A* **449–451,** 42–45.

6. Assael MJ *et al.* (2006) Reference data for the density and viscosity of liquid aluminum and liquid iron. *J. Phys. Chem. Ref. Data* **35**, 285–300.

7. Iida T, Guthrie RIL (1988) *The physical properties of liquid metals* (Clarendon Press, Oxford).

8. Assael MJ *et al.* (2010) Reference data for the density and viscosity of liquid copper and liquid tin. *J. Phys. Chem. Ref. Data* **39**, 033105.

9. Brillo J, Egry I, Ho I (2006) Density and Thermal Expansion of Liquid Ag–Cu and Ag–Au Alloys. *Int. J. Thermophys.* **27**, 494-506.

10. Kehr M, Schick M, Hoyer W, Egry I (2008) *High Temperatures-High Pressures* **37**, 361–369.

11. Paradis PF, Rhim WK (2000) Non-contact measurements of thermophysical properties of titanium at high temperature. J. Chem. Thermodyn. 32, 123–133.

12. Vinet B, Garandet JP, Cortella L (1993) Surface tension measurements of refractory liquid metals by the pendant drop method under ultrahigh vacuum conditions: Extension and comments on Tate’s law. *J. Appl. Phys.* **73**, 3830–3834.

13. Egry I (1999) Structure and properties of levitated liquid metals. *J. Non-Cryst. Solids* **250–252**, 63.

14. Okada JT, Ishikawa T, Watanabe Y, Paradis PF, Watanabe Y, Kimura K (2010)Viscosity of liquid boron. *Phys. Rev. B* **81**, 140201(R).
